# Intraoperative Post Partum Hemorrhage in a Patient with Dengue Fever

**DOI:** 10.12669/pjms.38.1.4519

**Published:** 2022

**Authors:** Usama Ahmed, Asiyah Aman

**Affiliations:** 1 Dr. Usama Ahmed, Fellow Pain Medicine, Department of Anaesthesiology, The Aga Khan University, Karachi, Pakistan; 2 Dr. Asiyah Aman, Assistant Professor, Department of Anaesthesiology, The Aga Khan University, Karachi, Pakistan

**Keywords:** Dengue, Blood Coagulation Disorders, Transfusion, Anesthesia, Obstetrics

## Abstract

A 33 year old obstetric patient with mild fever of undiagnosed etiology underwent emergency caesarean section under general anesthesia. She had platelet count of 98,000 per microliter and increased APTT of 37.8 s at the time of surgery. After uneventful anesthetic induction and delivery of fetus, slow and oozing type of bleeding led to massive hemorrhage. Patient remained vitally stable throughout perioperative phase and was extubated. Next day, patient’s dengue IgM antibody was reported positive. Neonate was well and his dengue test was negative. Pregnant women are at high risk of developing severe complications of dengue fever with unclear mechanisms related to impaired coagulation. Regional anesthesia may not have safe outcome due to dengue infection.

## INTRODUCTION

According to World Health Organization, Dengue virus infects more than 50 million annually of which more than 15000 die. September to November is the most critical period for the dengue fever endemic in Pakistan. The gravity of the problem related to Pakistan is so severe that 34.75% of patients presenting with the acute febrile illness have Dengue fever.^1^ According to the largest retrospective study of Dengue infection during pregnancy in the Pakistani population, maternal mortality rate was 7% which is quite high.^2^

Dengue virus belongs to the family Flaviviridae and genus Flavivirus. Aedes Aegypti is a vector of the disease. The incubation period is 3–8 days. Symptoms of disease are severe headache, joint and muscle discomfort, swollen glands, nausea, vomiting, and pain behind the eyes. Dengue Fever grades from mild to severe.

Pregnant women are at a higher risk of developing severe complications of the disease.^3^ We report case of a mild maternal dengue fever which led to hemorrhagic complications during emergency caesarean section.

## CASE REPORT

A 33 Years old un-booked parturient at 36th week of gestation came to emergency labor room of a tertiary care health institute of Karachi with chief complaint of decreased fetal movements. She had fever for five days associated with headache and generalized body aches. She had no known comorbidities and physical examination didn’t show focal findings other than fever. Surgical history included previous one Caesarean section which was uneventful. Socioeconomic history revealed that her residence was in an area of Karachi where Dengue Fever was endemic.

Labs were sent upon admission. Her CBC (Hb: 11.8g/dl, HCT: 37.2%, TLC: 4500, Platelets: 98,000/µL) revealed thrombocytopenia. Among the coagulation profile (PT: 10.3, INR: 1.0, APTT: 37.8) only APTT was abnormal. LDH was 341 IU/ml. Procalcitonin was 0.3ng/ml. Liver enzymes and kidney function tests were within normal limits. Malaria and typhoid screening were negative. Among her obstetric findings, CTG was non-reassuring.

We decided to give general anesthesia to the patient because of thrombocytopenia and fever. Rapid sequence induction was done. After delivery of fetus, the patient went into post-partum hemorrhage. Total blood loss was 3.5 Liters over a prolonged perioperative duration of 3.5 hours. Blood pressures were recorded non-invasively from induction till emergence. Four packed cells, four fresh frozen plasma and four platelets were transfused along with Lactated Ringer as maintenance fluid. Patient remained haemodynamically stable throughout the anesthesia maintenance. Hourly urine output was adequate. 3mg Dinoprostone rectal suppository was also administered. Tranexamic acid was loaded at a dosage of 10mg/Kg. Arterial blood gases parameters were normal at the end of surgery. The patient was extubated and remained stable in PACU.

Next day, the patient’s dengue IgM antibody was reported positive. Platelets dropped further to 48,000 per microliter on the first post-operative day. There was no bleeding after the operative phase. Patient was discharged from the hospital after four days. Platelets became normal on the ninth post-operative day. Neonate was well and his dengue test was negative.

## DISCUSSION

Existing literature regarding obstetric anesthetic management of patients with dengue fever is in the form of many case reports but no guidelines exist regarding anesthetic management of such patients. Therefore, indirect deductions can be made from literature till specific data becomes available. Pouliot SH et al conducted a systematic review of 30 published studies about the dengue virus during pregnancy. Of note, there was a 44% incidence of cesarean deliveries and various adverse outcomes were noted including hemorrhage.^4^ Hemorrhage due to dengue can range from superficial petechiae to life-threatening bleeding. There are problems with platelets (qualitative and quantitative) coexistent with coagulopathy and vasculopathy.

Thrombocytopenia occurs due to bone marrow suppression and peripheral destruction due to complement activation. It is also considered a marker of severity of the disease. There is an imbalance between clotting and fibrinolytic systems. Low fibrinogen levels and prolonged aPTT are the culprits for coagulopathy.^5^ Thromboelastography (TEG) analyses of dengue patients have revealed coagulation factor deficiency as the major abnormality followed by platelet dysfunction and primary fibrinolysis.^6^ Our obstetric setup did not have TEG facility to validate these findings.

There is conflicting evidence from literature in relation to our patient’s platelet count of 98,000/µL in terms of blood loss. They were adequate enough for neuraxial blockade but owing to increased aPTT and possible qualitative platelet defects (due to suspected dengue), neuraxial block was not opted. Platelet counts have been reported to be consistently unreliable in predicting bleeding.^7^ This is of concern for anesthesiologists as well as obstetricians. None of the case reports as per literature search matched our scenario as they had lower platelet counts and their reported bleeding was not significant.

Coagulopathy is a relative contraindication for spinal anesthesia and epidural hematoma is one of the serious complications. Therefore, we opted for General Anesthesia for this patient. In 2006, Chabbra A et al highlighted the case of a parturient who had excessive bleeding from the wound margins during the surgery.^8^ They advocated general anesthesia to prevent any adverse effects from regional anesthesia. Singh S et al have described the use of platelets to hack window for spinal anesthesia for surgery in an undiagnosed case of dengue fever in obstetric patient having a platelet count of 60 × 109/L with signs of dengue fever.^9^ No guidelines exist regarding regarding laboratory work-up for the perioperative period in pregnant patients with fever in dengue endemic areas. We recommend these to be devised.

Our patient had a massive post-partum hemorrhage despite her dengue was mild in severity. Massive hemorrhage is defined as blood loss in excess of one circulating volume within a 24-hour period. Other criterias are: Loss of 50% of blood volume within 3-h period, blood loss exceeding 150ml/min, blood loss requiring platelet and plasma transfusion. Postpartum Hemorrhage (PPH) is defined as loss of more than 500 ml or 1,000 ml of blood within the first 24 hours following childbirth which can occur intraoperatively as well.

There is a lack of evidence-based guidelines for transfusion of blood products in patients with dengue fever. Prophylactic platelet transfusions aren’t recommended because of fluid overload and transfusion related side effects. There is also decreased survival of transfused platelets due to non-discriminative immune-mediated destruction. In life-threatening bleeding, cryoprecipitate is recommended as it increases platelet function of both: donor and recipient. Recombinant Activated Factor VII use has also yielded good results to counteract bleeding.^10^ We neither used cryoprecipitate nor factor VII as bleeding was controlled after FFP transfusions.

Literature is silent about the duration of surgery in dengue fever, which can be prolonged in case of massive hemorrhage. Our decision was appropriate as the surgery lasted for 3.5 hours which could not have been possible in spinal anesthesia.

## CONCLUSION

Anesthesiologists and obstetricians must be cautious in the management of patients with clinical features of dengue in endemic areas or travel history from such areas. Qualitative and quantitative platelet defects, coagulopathy and vasculopathy due to the disease can lead to life-threatening hemorrhage and the duration of surgery can be prolonged. Regional Anesthesia may not have safe outcome in such cases. An individualized approach for perioperative anesthetic management is best.
